# Supplementary material for: Implementing evidence ecosystems in the public health service: Development of a framework for designing tailored training programs
Source: PLoS One. 2024 Apr 18;19(4):e0292192. doi: 10.1371/journal.pone.0292192 (PMC11025971; doi:10.1371/journal.pone.0292192)
Supplement: S2 Table — (DOCX) [file pone.0292192.s002.docx]

***Table S2.*** *Search strategy for the database PUBMED*

| ((((Public Health Administration[MeSH Terms] OR Public Health Practice[MeSH Terms] OR Personnel Turnover[MeSH Terms] OR Public Health Systems Research[MeSH Terms] OR Schools, Public Health[MeSH Terms]) AND (train*[Title] OR educat*[Title] OR postgrad*[Title] OR post-grad*[Title] OR specialt*[Title]))) OR ((public health[Title] OR health authori*[Title] OR health department*[Title]) AND ((Health Workforce[MeSH Terms] OR Capacity Building[MeSH Terms] OR Personnel Management[MeSH Terms] OR Education, Public Health Professional[MeSH Terms]) OR (train*[Title] OR educat*[Title] OR postgrad*[Title] OR post-grad*[Title] OR specialt*[Title] OR (capacity[Title] AND building[Title]) OR specialty[Title] OR workforce*[Title] OR manpower[Title] OR competenc*[Title] OR abilit*[Title] OR skill*[Title] OR curricul*[Title] OR learning goals[Title] OR residenc*[Title]))) OR (((public health[Title] OR health authori*[Title] OR health department*[Title]) OR (public health workforce[Title/Abstract] OR epidemiolog*[Title/Abstract] OR Public health special*[Title/Abstract])) AND (capacity building[Title/Abstract] OR capacity building[MeSH Terms] OR (capacity[Title/Abstract] AND building[Title/Abstract]) OR specialt*[Title] OR postgrad*[Title/Abstract] OR post-grad*[Title/Abstract] OR program*[Title/Abstract]) AND (educat*[Title/Abstract] OR train*[Title/Abstract]))) AND (((Switzerland* OR Swiss* OR Schweiz*) OR (Aargau OR Appenzell Outer-Rhodes OR Appenzell Ausser-Rhoden OR Appenzell Inner-Rhodes OR Appenzell Inner-Rhoden OR Basel OR Basel District OR Basel Landschaft OR Bern OR Bern OR Fribourg OR Freiburg OR Geneva OR Genf OR Glarus OR Glarus OR Grisons OR Graubünden OR Jura OR Jura OR Lucerne OR Luzern OR Neuchâtel OR Neuenburg OR Nidwalden OR Obwalden OR Schwyz OR Schaffhausen OR Solothurn OR St Gallen OR St. Gallen OR Ticino OR Tessin OR Thurgau OR Uri OR Vaud OR Waadt OR Valais OR Wallis OR Zug OR Zurich OR Zürich)) OR (UK OR united kingdom OR Great Britain OR Wales OR Scotland OR England OR northern ireland OR welsh OR scottish) OR ((Austria* OR Österreich*) OR (Burgenland OR Carinthia OR Kärnten OR Lower Austria OR Niederösterreich OR Upper Austria OR Oberösterreich OR Salzburg OR Styria OR Steiermark OR Tyrol OR Tirol OR Vorarlberg OR Vienna OR Wien)) OR ((Netherland OR dutch) OR (Drenthe OR Flevoland OR Friesland OR Gelderland OR Groningen OR Limburg OR North Brabant OR North Holland OR Overijssel OR south holland OR Holland OR Utrecht OR Zeeland OR Amsterdam)) OR ((Germany OR German OR Deutschland OR deutsch) OR (Baden-Wuerttemberg OR Bavaria OR Berlin OR Brandenburg OR Bremen OR Hamburg OR Hesse OR Lower Saxony OR Mecklenburg-Western Pomerania OR North Rhine-Westphalia OR Saarland OR Saxony OR Saxony-Anhalt OR Schleswig-Holstein OR Thuringia OR Thüringen OR Sachsen-Anhalt OR Sachsen OR Nordrhein-Westfalen OR Mecklenburg Vorpommern OR Niedersachsen OR Hessen OR Bayern OR Baden-Württemberg)))  AND (2011[Date - Publication] : 3000[Date - Publication]) |
| --- |
