# Supplementary material for: Implementing evidence ecosystems in the public health service: Development of a framework for designing tailored training programs
Source: PLoS One. 2024 Apr 18;19(4):e0292192. doi: 10.1371/journal.pone.0292192 (PMC11025971; doi:10.1371/journal.pone.0292192)
Supplement: S3 Table — (DOCX) [file pone.0292192.s003.docx]

**Table S3.** Search strategy for the database LIVIVO

| MESH=(Public Health Administration OR Public Health Practice OR Personnel Turnover OR Public Health Systems Research OR Schools, Public Health OR Health Workforce OR Capacity Building OR Personnel Management OR Education, Public Health Professional)  AND TI=(public health OR health authori* OR health department* OR epidemiolog* OR public health specialt*) AND TI=(train* OR educat* OR postgrad* OR post-grad* OR specialt* OR (capacity AND building) OR specialty OR workforce* OR manpower OR competenc* OR abilit* OR skill* OR curricul* OR learning goals OR residenc*) AND TI=((((Switzerland* OR Swiss* OR Schweiz*) OR (Aargau OR Appenzell Outer-Rhodes OR Appenzell Ausser-Rhoden OR Appenzell Inner-Rhodes OR Appenzell Inner-Rhoden OR Basel OR Basel District OR Basel Landschaft OR Bern OR Bern OR Fribourg OR Freiburg OR Geneva OR Genf OR Glarus OR Glarus OR Grisons OR Graubünden OR Jura OR Jura OR Lucerne OR Luzern OR Neuchâtel OR Neuenburg OR Nidwalden OR Obwalden OR Schwyz OR Schaffhausen OR Solothurn OR St Gallen OR St. Gallen OR Ticino OR Tessin OR Thurgau OR Uri OR Vaud OR Waadt OR Valais OR Wallis OR Zug OR Zurich OR Zürich)) OR (UK OR united kingdom OR Great Britain OR Wales OR Scotland OR England OR northern ireland OR welsh OR scottish) OR ((Austria* OR Österreich*) OR (Burgenland OR Carinthia OR Kärnten OR Lower Austria OR Niederösterreich OR Upper Austria OR Oberösterreich OR Salzburg OR Styria OR Steiermark OR Tyrol OR Tirol OR Vorarlberg OR Vienna OR Wien)) OR ((Netherland OR dutch) OR (Drenthe OR Flevoland OR Friesland OR Gelderland OR Groningen OR Limburg OR North Brabant OR North Holland OR Overijssel OR south holland OR Holland OR Utrecht OR Zeeland OR Amsterdam)) OR ((Germany OR German OR Deutschland OR deutsch) OR (Baden-Wuerttemberg OR Bavaria OR Berlin OR Brandenburg OR Bremen OR Hamburg OR Hesse OR Lower Saxony OR Mecklenburg-Western Pomerania OR North Rhine-Westphalia OR Saarland OR Saxony OR Saxony-Anhalt OR Schleswig-Holstein OR Thuringia OR Thüringen OR Sachsen-Anhalt OR Sachsen OR Nordrhein-Westfalen OR Mecklenburg Vorpommern OR Niedersachsen OR Hessen OR Bayern OR Baden-Württemberg))))  AND PY=2011:2050 |
| --- |
