# Supplementary material for: Implementing evidence ecosystems in the public health service: Development of a framework for designing tailored training programs
Source: PLoS One. 2024 Apr 18;19(4):e0292192. doi: 10.1371/journal.pone.0292192 (PMC11025971; doi:10.1371/journal.pone.0292192)
Supplement: S4 Table — (DOCX) [file pone.0292192.s004.docx]

***Table S4.*** Overarching coding tree structure from the scoping review.

| 1. Context-related aspects of the qualification model  1.1 Duration and density  1.2 Professional credentialing  1.3 Program objectives  1.4 Recruitment and selection process  1.5 Stakeholder involvement  1.6 Resources  1.7 Setting  2. Content-related aspects of a qualification model  2.1 Integration of content into program  2.1.1 Horizontal Integration  2.1.2 Vertical Integration  2.2 Training & education forms  2.2.1 Teacher-centered training forms  2.2.2 Interaction of participants  2.2.3 Exposure to practice  2.3 Didactical concepts  2.4 Measurability and assessment  3. Translation into a qualification model  3.1 Workshops, seminars and trainings  3.2 Hospitation (e.g. internship) in PH-institutions  3.3 Master programs in public health  3.4 PhD-programs in public health  3.5 Integration of PH into medical curricula  3.6 Postgraduate/Trainee programs  3.7 Rotation model practice and science  3.8 Peer Mentoring  3.9 Labelling  4. Consolidation and further development  4.1 Piloting  4.2 Dissemination  4.3 Evaluation and quality assessment  4.3.1 Evaluation methods  4.3.2 Factors and barriers for program success  4.3.3 Reaction (Level 1)  4.3.4 Learning (Level 2)  4.3.5 Behavior (Level 3)  4.3.6 Results (Level 4)  4.3.7 ROI (Level 5)  4.4 Transferability  4.5 Continuous program development |
| --- |
