# Supplementary material for: Implementing evidence ecosystems in the public health service: Development of a framework for designing tailored training programs
Source: PLoS One. 2024 Apr 18;19(4):e0292192. doi: 10.1371/journal.pone.0292192 (PMC11025971; doi:10.1371/journal.pone.0292192)
Supplement: S5 Table — (DOCX) [file pone.0292192.s005.docx]

**Table S4**. Characteristics of the studies included in the scoping review.

| **First Author (Year)** | **Ref** | **Short title** | **Country** | **Qualification program, main focus** |
| --- | --- | --- | --- | --- |
| Baxter (2016) | (62) | A new infection trainee education programme from the Healthcare Infection Society | UK | Structured education program focusing on infection training and including infection prevention and control (IPC) |
| Bennetts (2012) | (73) | Continuing professional development for public health: an andragogical approach | UK | Continuing professional development (CPD) |
| Buunaaisie (2018) | (71) | Employability and career experiences of international graduates of MSc Public Health: a mixed methods study | UK | Master of Science Program (MSc) |
| Chastonay 2012) | (72) | Design, implementation and evaluation of a community health training program in an integrated problem-based medical curriculum | CH | 6-year longitudinal and multidisciplinary Community Health Program (CHP) |
| Cheetham (2018) | (51) | Embedded research: a promising way to create evidence-informed impact in public health? | UK | Embedded research (ER)  co-located between academia and local health authority (LHA) |
| Currie (2020) | (52) | Public Health Ausbildung in Österreich. Ein Überblick | UK, AU | Specialty public health training |
| Dey (2019) | (63) | The United Kingdom Field Epidemiology Training Programme: meeting programme objectives | UK | Field epidemiology training programmes (FETP) |
| Diem (2016) | (64) | Prevention and control of noncommunicable diseases through evidence-based public health: implementing the NCD 2020 action plan | AT | Capacity building efforts focusing on knowledge translation for non-communicable diseases (NCD) |
| Dorner (2014) | (53) | Long-Term Evaluation of a Course on Evidence-Based Public Health in the U.S. and Europe | AT | Postgraduate public health university courses and public health doctoral programs |
| Erwin (2014) | (54) | Long-Term Evaluation of a Course on Evidence-Based Public Health in the U.S. and Europe | US | Evidence-based public health course |
| Gerhardus (2017) | (74) | Public Health als anwendungsorientiertes Fach und Multidisziplin – „Forschendes Lernen“ als Antwort auf die Herausforderungen für Lehren und Lernen? | DE | Research-based learning approaches within a student-led research project |
| Gillam (2016) | (69) | Public health education in UK medical schools-towards consensus | UK | Consensus statement to support the development of learning environments for public health; public health rotation |
| Gray ( 2018) | (55) | Developing the public health workforce: training and recognizing specialists in public health from backgrounds other than medicine: experience in the UK | UK | Multidisciplinary training of senior public health specialists;  Registry for public health specialists |
| Harrison (2015) | (56) | The effect of using different competence frameworks to audit the content of a masters program in public health | UK | Competence frameworks in master public health programs |
| Heller (2017) | (57) | Open Online Courses in Public Health: experience from Peoples-uni | UK | Open Online Courses (OOCs) as open educational resources |
| Idler (2016) | (58) | Prevention and health promotion from theory to practice: The interprofessional MeMPE Summer University for students of Medicine, Master of Public Health and Epidemiology | DE | Interprofessional Seminar on prevention and health promotion in the course of a summer university |
| Jansen (2013) | (59) | A masterclass to teach public health professionals to conduct practice-based research to promote evidence-based practice: a case study from The Netherlands | NL | Masterclass for public health professionals focusing on practice-based research skills |
| Könings (2018) | (60) | Is blended learning and problem-based learning course design suited to develop future public health leaders? | NL | Problem-based, blended learning method in a virtual learning environment |
| McCulloch (2014) | (67) | Developing capacity in field epidemiology in England | UK | Field epidemiology training programmes (FETP) |
| Peik (2016) | (65) | Comparison of public health and preventive medicine physician specialty training in six countries: Identifying challenges and opportunities | US | Descriptive profiles of national training demographics and structures of Public health and preventive medicine (PHPM) specialties |
| Ramsay (2014) | (61) | The Structured Operational Research and Training Initiative for public health programmes | CH | Structured operational research and training initiative |
| Salway (2013) | (76) | Improving capacity in ethnicity and health research: report of a tailored programme for NHS Public Health practitioners | UK | Tailored program focusing on research capacity development |
| Smith (2015) | (68) | Principles of all-inclusive public health: developing a public health leadership curriculum | UK | Curriculum development focusing on public health leadership skills |
| Tran (2017) | (75) | In-house peer supported literature search training: a public health perspective | UK | Peer supported literature search training course; Knowledge and library service (KLS) |
| Turner-Wilson (2017) | (70) | Can nurses rise to the public health challenge? How a novel solution in nurse education can address this contemporary question | UK | Public health improvement theme running throughout an undergraduate nursing curriculum |
